# Spots on the Sun

**Published:** 1890-10

**Authors:** 


### SPOTS ON THE SUN.

Scientists tell us that the sun is a body in a state of active incandescence; that the spots which are visible on its face are volumes of smoke arising from beds of carboniferous matter in a state of combustion.

These spots, as vast and dismal as they are, appear to the earth's inhabitants quite insignificant. Certain it is, they neither perceptibly diminish the sun's light nor its heat. It is only on close inspection they assume importance; and the closer they are scrutinized the blacker they are.

So with "spots" on one's character; it must be a large and black "spot" indeed to overshadow all the good in one's life; yet, generally, the more closely they are scrutinized the blacker they seem.

So, in communities. There are black sheep in all flocks, they say. The presence of a few unworthy persons in a State Medical Association should not, does not, detract from the merits of the body as a whole; it is only when the characters of those who are unworthy are investigated singly and without reference to the society, that they loom up dark and dismal. They do not perceptibly diminish the lustre of the body to which they belong; do not detract in the least from its dignity, its ability or its honor.

We have said on former occasions that the presence of an element of unworth in the State Association leavens the loaf and brings discredit upon the whole. This is only partly true, and applies to the laity only. Scientifically the Association is unaffected by their presence.

On several occasions, the JOURNAL, inspired by the hope of inciting to a higher standard of qualification for membership, has pointed out where, in its judgment, mistakes had been made, and persons admitted to membership who are really unworthy of the Association, It is to be hoped that we all may profit by experience.

In the Texas State Medical Association are men of distinguished ability, and of untarnished reputation; in fact, the great majority of its members are men of a very superior order; and the Association stands to-day in the very foremost rank of medical and scientific bodies, and shines as a star of first magnitude in the medical firmament. That there are a few isolated cases which constitute the exception should not, and does not detract in the least from its brilliancy. Its works speak for themselves; they have given the Association a front place even in the estimation of our European confreres who were for a long time loth to admit that any good could come out of Nazareth.

The JOURNAL'S sole aim is to advance and elevate true medicine, to commend the good, to denounce the false and bad. To the promotion of the best interests of legitimate medicine in our adopted State our best endeavors are conscientiously dedicated, and we claim, and without fear of successful contradiction, that the policy of the JOURNAL has largely contributed to the bettered state of the Association, the better enforcement of its laws and the quicker and more satisfactory dispatch of business; as well as to the greatly increased membership. By personal solicitation, as well as through the JOURNAL many good men have been induced to join us in the good work.

